# Novel Spontaneous Deletion of Artemis Exons 10 and 11 in Mice Leads to T- and B-Cell Deficiency

**DOI:** 10.1371/journal.pone.0074838

**Published:** 2013-09-17

**Authors:** Christian Barthels, Jacek Puchałka, Tomas Racek, Christoph Klein, Thomas Brocker

**Affiliations:** 1 Institute for Immunology, Ludwig-Maximilians-Universität, München, Germany; 2 Dr. von Hauner Children’s Hospital, Department of Pediatrics, Ludwig-Maximilians-Universität, München, Germany; Université Libre de Bruxelles, Belgium

## Abstract

Here we describe a novel, spontaneous, 4035 basepairs long deletion in the DNA cross-link repair 1C (*Dclre1c*)-locus in C57BL/6-mice, which leads to loss of exons 10 and 11 of the gene encoding for Artemis, a protein involved into V(D) J-recombination of antigen receptors of T and B cells. While several spontaneous mutations of Artemis have been described to cause SCID in humans, in mice, only targeted deletions by knockout technology are known to cause the same phenotype so far. The deletion we observed causes a loss of Artemis function in the C57BL/6 strain and, consequently, the absence of T and B cells, in presence of normal numbers of NK cells and cells of the myeloid lineage. Thus, for the first time we present T^-^B^-^NK^+^ severe combined immunodeficiency (SCID) phenotype after spontaneously occurring modification of Artemis gene in mice. Our mouse model may serve as a valuable tool to study mechanisms as well as potential therapies of SCID in humans.

## Introduction

Severe combined immunodeficiency (SCID) comprises a heterogeneous group of rare genetic disorders characterized by absence of T and B cell immune response. The B cell defects can hereby be caused either indirectly, due to missing T cell help, or directly, by intrinsic B cell deficiencies. Affected patients suffer from life-threatening recurrent infections, chronic diarrhea and failure to thrive. Treatments include hematopoietic stem cell transplantation or gene therapy, to correct gene defects [1].

SCID phenotypes are classified according to the presence or absence of T and/or B cells with selective lack of T cells being a T^-^B^+^NK^+^ SCID or the combined absence of T and B cells in the presence of NK cells being T^-^B^-^NK^+^ SCID [2]. SCID phenotypes can be caused by a variety of genetic defects affecting T and or B cell development in humans as well as in mice [3,4]. Common to all SCID phenotypes with B and T cell deficiencies is a defect in proteins controlling the TCR and BCR V(D) J-recombination machinery from germline encoded variable (V), diversity (D) and joining (J) gene segments [5].

In the first stage of this process the products of recombination activating genes 1 and 2 (RAG1 and RAG2) introduce double strand breaks (DSB), which are then sealed by ubiquitously expressed factors of the non-homologous end joining (NHEJ) machinery consisting of at least 7 proteins. Subsequently, the Ku70/Ku80 complex binds to the DSB and recruits DNA-dependent protein kinase (DNA-PKcs) [6,7]. Artemis then binds and is activated through phosphorylation by DNA-PKcs. Activated Artemis then opens the hairpin structures [8], which are further processed, ligated and sealed by NHEJ factors XRCC4 and DNA-LigIV [9]. This process is promoted by XLF [10], the last protein discovered to play a role in NHEJ.

Mutations in many of these genes have been described to cause SCID-phenotypes of different severity in humans. Defects in RAG1 or RAG2 lead either to a T^-^B^-^NK^+^ SCID, caused by null mutations, or to Omenn syndrome with residual B and T cell development caused by hypomorphic mutations [11]. Mutations in the Artemis gene *DCLRE1C* are found in populations all over the world with an accumulation of a specific mutation in the Athabascan speaking native Americans, who have a high incidence of SCID (called SCID-A in this case) [12]. This and other *DCLRE1C*-mutations cause a phenotype similar to the one observed in RAG-deficiencies. In addition, these patients also present with an increased sensitivity of the cells to ionizing radiation (RS-SCID) [13].

DNA-LigIV defects resemble very much XLF-deficiencies causing T^-^B^-^NK^+^SCID or Omenn syndrome, accompanied among other defects by microcephaly and growth retardation [14,15]. Also mutations in DNA-PKcs, first described in horses, dogs and mice as SCID [16], do occur in humans [17,18] and are characterized by T^-^B^-^NK^+^SCID phenotype as well as by radiosensitivity [18].

Knockout mice have been generated for all components of the V(D)J recombination machinery and faithfully phenocopy the defects described above [19-24]. XRCC4- and DNA-LigVI-deficiencies caused lethality of the embryo [25,26], while hypomorphic mutations showed the expected SCID phenotype [27,28]. In contrast to humans, where many spontaneous mutations have been described for several components of V(D)J recombination machinery, only one spontaneous mutation has been described in mouse until now [16]. But mutations mirroring human mutations have been introduced into the mouse genome successfully recapitulating the phenotype found in humans [28].

Here we describe a novel, spontaneous deletion of exon 10 and 11 in the mouse *Dclre1c*-gene, resulting the corresponding truncated Dclre1c-mRNA and complete absence of mature B and T cells. This model faithfully resembles the phenotype of human mutations in this region of *Dclre1c* [29] and is therefore the first spontaneous mouse model for SCID-A. 

## Materials and Methods

### Animals

Mice were bred and housed at the animal facilities of the Institute for Immunology (LMU, Munich, Germany) and treated in accordance with established guidelines of the Regional Ethics Committee of Bavaria. Animal protocols were approved by local authorities.

### Flow Cytometry Analysis

Single cell suspensions were prepared from spleen, thymus and bone marrow from 5 to 10 week old mice. Red blood cells were lysed using ACK buffer for 5 min at RT. Where possible, 2x10^6^ cells were used for every staining with titered antibodies in PBS containing 2% FCS and 0.01% NaN_3_ (FACS buffer) for 20 min at 4°C in the dark. Cells were washed once and either used for direct acquisition on BD FACSCanto or fixed using 2% paraformaldehyde in FACS buffer and measured the next day. Dead cells were always excluded using Live/Dead Aqua (LifeTechnologies). Analysis was performed using FlowJo (Treestar).

### SNP Analysis

Homozygous mutant mice on C57BL/6-background were bred with C3H-mice and resulting F1 mice were used for an F2-intercross used for the analysis. F1 and F2 mice were analyzed for presence of B and T cells in blood. DNA was prepared from tails tips of animals without B and T cells using DNeasy Blood & Tissue kit (Qiagen) and DNA was frozen at -20°C until SNP analysis. For the SNP analysis the Mouse LD Linkage chip (Illumina) was used and procedure was performed according to manufacturer’s protocols. This chip contains 377 SNPs of which 244 were informative in our cross. Data were analyzed using DChip software.

### Cell sorting using magnetic beads

Thymi were taken out, homogenized and red blood cells were lysed using ACK buffer for 5 min at RT. Cells were stained with biotinylated antibodies directed against CD4 and CD8. Cells were depleted using streptavidin-microbeads (Miltenyi) according to manufacturer’s protocol. Purity was ascertained using flow cytometry and was routinely between 90% and 95%

### Isolation of RNA and preparation of cDNA

Sorted cell were resupended in Trizol and mRNA was isolated using RNeasy kit (Qiagen) according to manufacturer’s protocol. 2.5 µg of mRNA was transcribed into cDNA using SuperScript^®^ Vilo cDNA synthesis kit (Invitrogen).

### Next Generation Sequencing

Genomic DNA from SCID affected mice and corresponding parent animals was used for construction of exome libraries using SureSelect XT Mouse All Exon kit (Agilent) capturing 49.6 Mb of mouse exon sequences. Bar-coded libraries were sequenced using SOLiD 5500 next generation sequencing platform (LifeTechnologies) to an average coverage depth of 80x. Reads were aligned to the murine reference genome (version mm9) using LifeScope Analysis Suite 2.5.1 (LifeTechnologies) (using standard parameters) and the lane data for each sample merged using SAMtools version 1.18 (PubMed: 19505943). Subsequently, the PCR duplicates were removed and the sequencing depth within Dclre1c locus extracted (by applying ‘samtools rmdup’ and ‘samtools depth’ commands, respectively). The sequencing data is available at the European Nucleotide Archive (http://www.ebi.ac.uk/ena/). The accession number is PRJEB4418.

### PCR analysis of Artemis gene

DNA was prepared from mouse-tail tips using the DNeasy Blood & Tissue kit (Qiagen) according to manufacturer’s protocol. DNA was amplified using the following primers: p1f CACCATCTCACAACGGACAG, p2f CCCACTTCTACGATGACCCTGAG, p2r CTCAGGGTCATCGTAGAAGTGGG, p3f AGCTCCCGTTGCTCTCTCAG, p3r CTGAGAGAGCAACGGGAGCT, p4f TGAAGTGCTCAGGTCCCGTG, p4r CACGGGACCTGAGCACTTCA, p5f CCGTTCGGCAACAGCTTCTC, p5r GAGAAGCTGTTGCCGAACGG, p6f CTCTAAGTCCAGCAGGAGG, p7f CCGCTGTGTGCTATTTGACTC, p7r GAGTCAAATAGCACACAGCGG, p8f CCATTACCTTCTCCTCCTCC, p9r CACATTCACTGGGCAGATGT. Resulting PCR-products were analyzed with ethidiumbromide containing agarose-gel electrophoresis.

### Statistics

Significance was determined using the Student’s T test and defined as follows: *p<0.05, **p<0.01 and ***p<0.001. Bar graphs show average +/- SD from combined experiments and group sizes as indicated in the figure legends.

## Results

**Figure 1:**
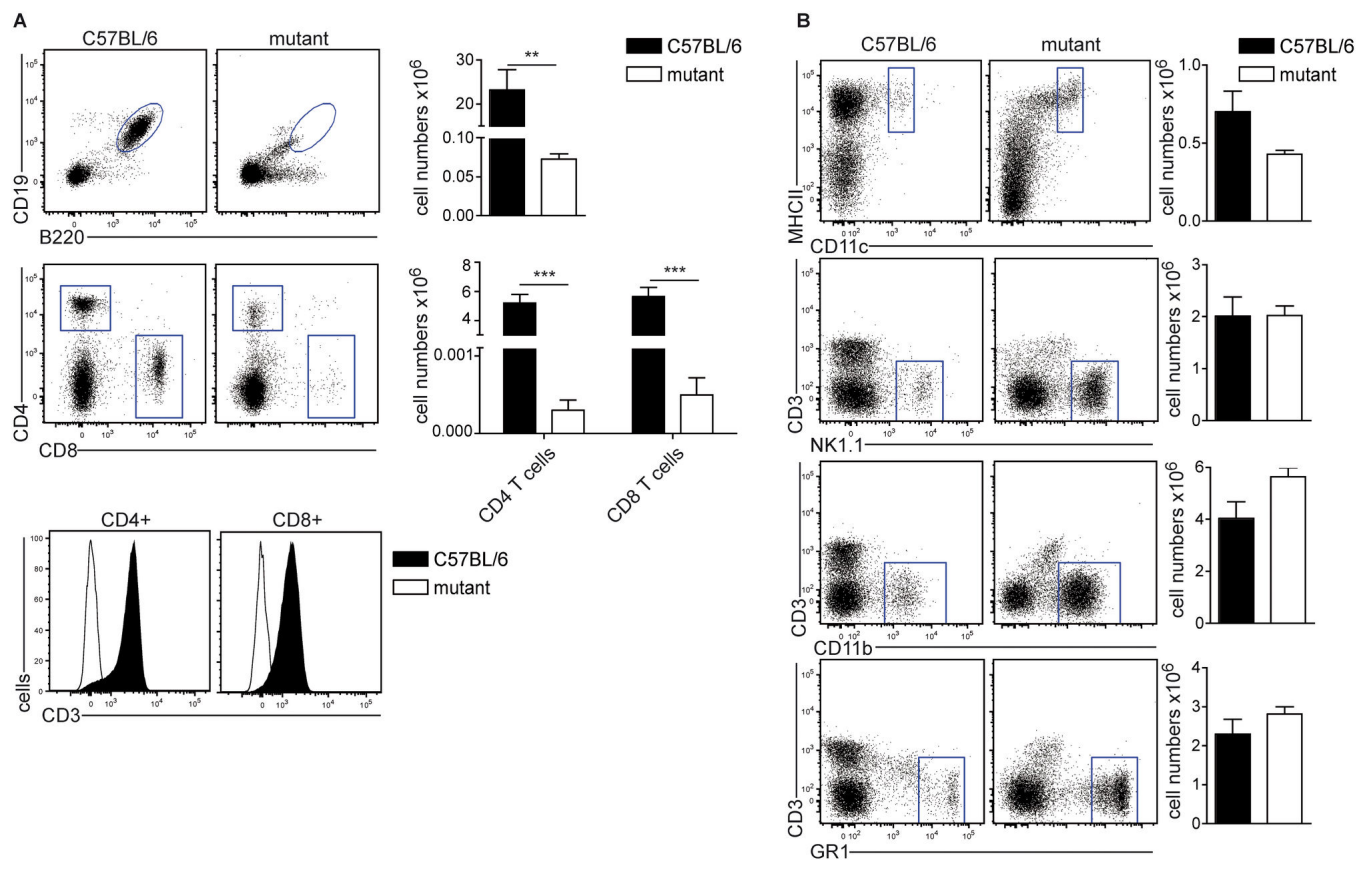
Initial characterization of cell subsets in the spleens of mutant mice. (A) Single cell suspensions of spleens were stained for B cells (CD19^+^B220^+^) or T cells (CD4^+^ or CD8^+^), which were further analyzed for expression of CD3 (lower panel). Cells were gated on live lymphocytes. (B) Single cell suspensions of spleens were stained with the indicated antibodies to identify DC (CD11c^+^MHCII^+^), NK cells (NK1.1 ^+^ CD3^-^), granulocytes (Gr1 ^+^ CD3^-^) and CD11b^+^ monocytes. FACS-plots and statistics are representative results from one out of six experiments with similar outcome (n=3 mice per group). Bar graphs represent mean number of live cells ± SD in the respective gate.

During routine phenotyping we detected in our mouse colony animals that had lost CD4^+^ and CD8^+^ T cells. To characterize this phenotype further, we analyzed different immune cell subsets in the spleens of these mice. These analyses revealed that besides CD4^+^ and CD8^+^ T cells also B cells were missing (Figure 1A), while the cell numbers of CD11c^+^MHCII^+^ dendritic cells, CD3^-^NK1.1^+^ NK cells, CD11b^+^ monocytes and Gr1^+^ granulocytes were not significantly altered (Figure 1B). The few CD4+ or CD8+ cells in the spleen of mutant mice were negative for CD3ε, indicating that they were not T cells (Figure 1A).

**Figure 2:**
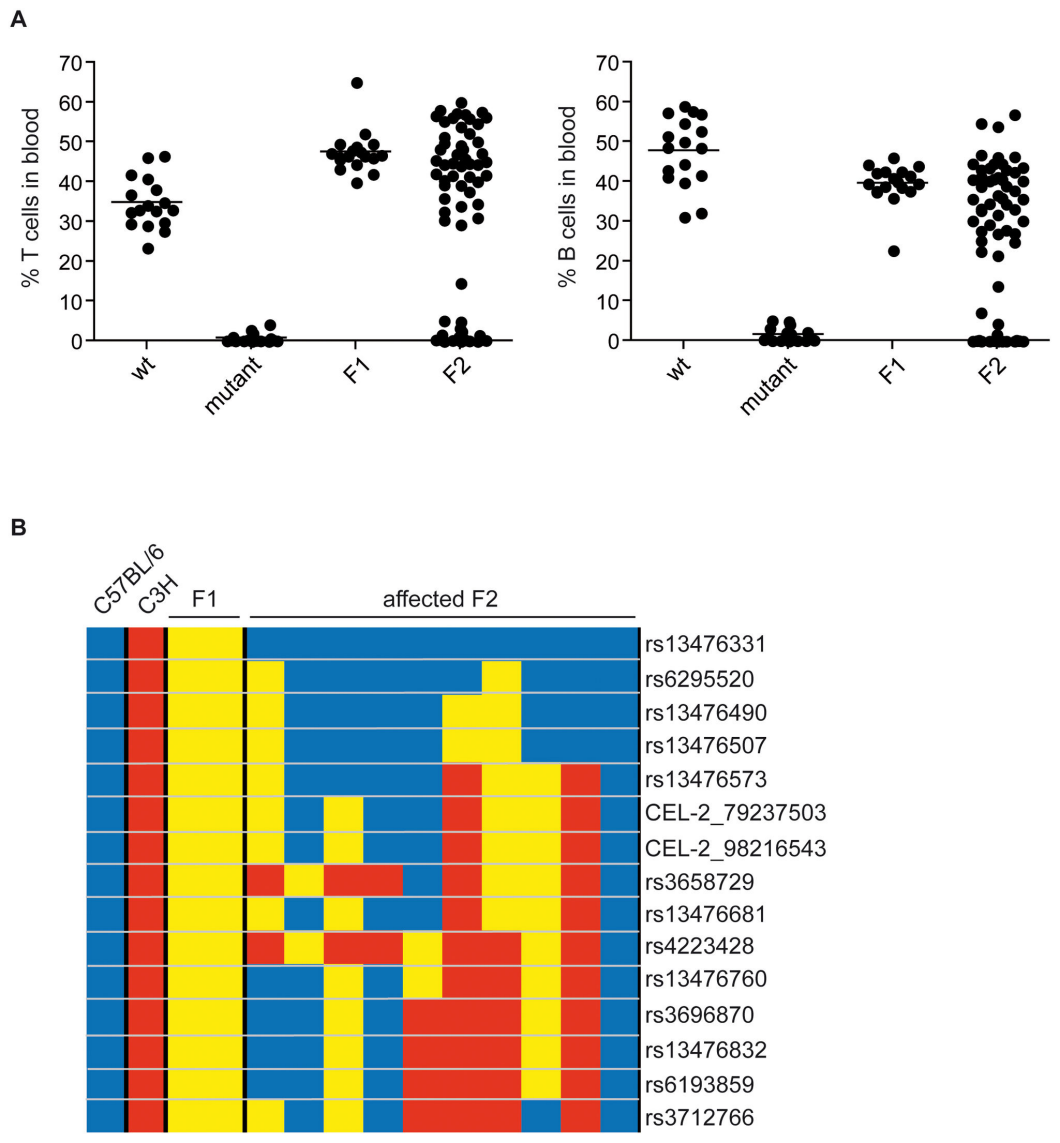
Single nucleotide polymorphism (SNP) analysis of mutant mice. (A) Mutant mice on C57BL/6 background were crossed to C3H and the offspring (F1) was used for an intercross yielding F2 animals. Blood of these animals was analyzed for the percentage of T cells (TCR-β ^+^ DX5^-^) and B cells (CD19^+^MHCII^+^). F2 animals were grouped according to the presence (unaffected) or absence (affected) of T and B cells and affected animals were used for SNP-analyses. (B) Graphical representation of SNP distribution in randomly selected F2 and control animals. SNPs derived from C57BL/6 are in blue, SNPs from C3H are in red and heterogenous SNPs are yellow. Shown are only informative SNPs at the beginning of chromosome 2. The chip contained 377 SNP of which 244 were informative in our cross. In total we performed SNP-analyses from 57 F2 animals as well as from 6 F1, C57BL/6 and C3H control animals each.

To identify the genomic region causing this phenotype, the mutant mouse strain, which was on C57BL/6-background, was crossed to C3H-mice and the resulting F1-offspring was used for an F2-intercross. Both, F1- and F2-generations were monitored for the presence of T and B cells (Figure 2A) as well as NK cells (data not shown). While we could not detect B or T cells in the mutant, all F1 animals showed normal frequencies of B and T cells in peripheral blood (Figure 2A). In approximately 25% of all F2 animals (68 out of 291 animals), B and T cells were lacking, a frequency that clearly indicates an autosomal recessive inheritance.

DNA of these affected F2 mice was used to perform a chip-based SNP-analysis. SNP rs13476331 at the beginning of chromosome 2 was the only SNP derived from C57BL/6, which correlated with occurrence of T and B cell-deficiency in all F2 animals (Figure 2B). We concluded that the gene carrying the mutation responsible for the observed phenotype must be located in this region between the beginning of chromosome 2 and the next not uniformly C57BL/6-derived SNP (rs6295520), resulting in a region of 43 Mbp containing 649 genes. One of these genes - *Dclre1c* (Artemis) is known to play a role in B and T cell development and its knockout causes SCID-phenotypes similar to our observed mutation. This correlation made *Dclre1c* an attractive candidate for the phenotype-causing gene.

**Figure 3:**
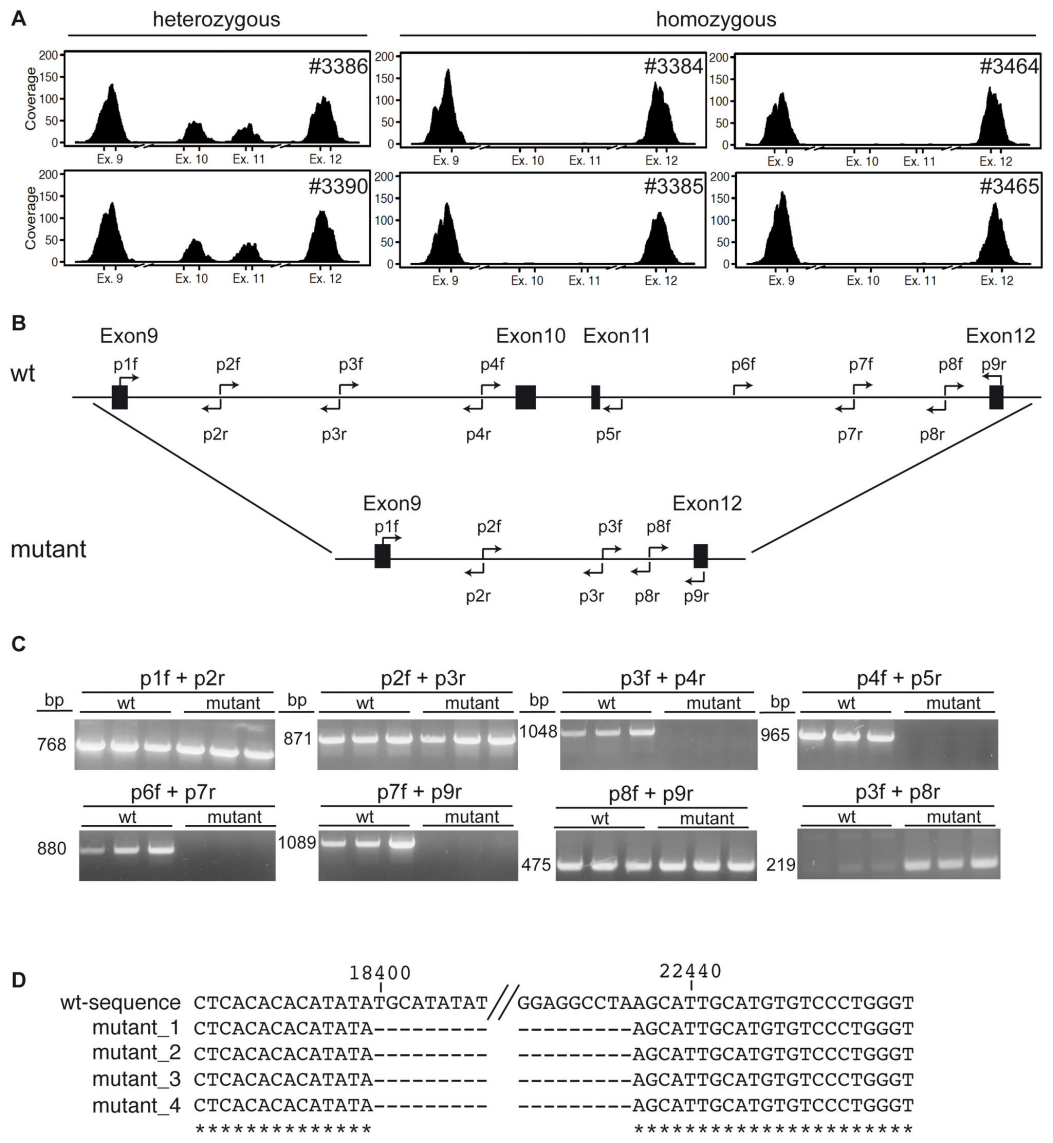
Sequencing of *Dclre1c-*gene. (A) Two heterogeneous and four homogeneous mutant mice were analyzed using next-generation-sequencing techniques. Displayed is the coverage of the bases in exons 9 to 12. (B) Graphical representation of the genomic organization of *Dclr1c* between exons 9 and 12. Exons are indicated by black boxes, while arrows indicate primer binding-sites. The upper row represents the organization in the wild-type genome while the lower row represents the gene in the mutant. (C) Results of PCR reactions with the indicated primer sets. DNA was visualized on ethidium bromide agarose gels. (D) Primer p3f and p8r were used to amplify DNA from mutant animals and the purified PCR products were used for Sanger DNA-sequencing. The resulting sequences were aligned to the *Dclre1c*-sequence according to the “Gene” database of NCBI. (*) indicates matching bases, (-) indicates gaps in the alignment. Shown is the 5´ beginning of the deletion (before //) as well as the 3´ end of the deletion (after //).

In parallel to the SNP-analysis we sequenced six mice using an exon-capture assay on the SOLiD platform. Two of these mice were heterozygous for the mutation, therefore showing no SCID, while the other four showed the phenotype and were homozygous for the mutation. Upon genome wide analysis, the coverage of the exons comprising the *Dclre1c*-gene was incomplete (Figure 3A) and did show a pattern, which fits to the presumed disease inheritance mode. Figure 3A shows the coverage (number each base was read) for exons 9 to 12 of *Dclre1c*. Although the coverage of *Dclre1* exons 9 and 12 was excellent in all animals, there were no reads in exons 10 and 11 in animals homozygous for the mutation. Furthermore, the coverage of these exons in animals heterozygous for the mutation was decreased as compared to the surrounding ones (Figure 3A).

For a more detailed analysis of this part of the gene, 9 primer pairs were designed spanning the whole region between exons 9 and 12 (Figure 3B upper panel). DNA from wild type animals (wt) as well as mutants was amplified using the primer combinations indicated in Figure 3B. Wt DNA yielded bands with all of these primer pairs while mutant DNA was only amplified using primers p1f/p2r, p2f/p3r and p8f/p9r indicating missing primer-binding sites in between (Figure 3B). A PCR using primers p3f and p8r resulted only in a product when DNA from mutant, but not wild type mice was used as template (Figure 3B). This PCR 200bp product was purified and directly used for DNA-sequencing. Resulting sequences of mutants were aligned to the wt-sequence of *Dclre1c* showing that mutant mice harbor a 4035bp-deletion between positions 18401 and 22436 of the *Dclre1c*-genomic sequence (Figure 3C). This deletion could also be detected on the mRNA-level, as PCR amplification using primers p1f and p9r of reverse transcribed mRNA from wt and mutant CD4^-^CD8^-^ thymocytes revealed length differences corresponding to the deletion of exons 10 and 11 (data not shown).

**Figure 4:**
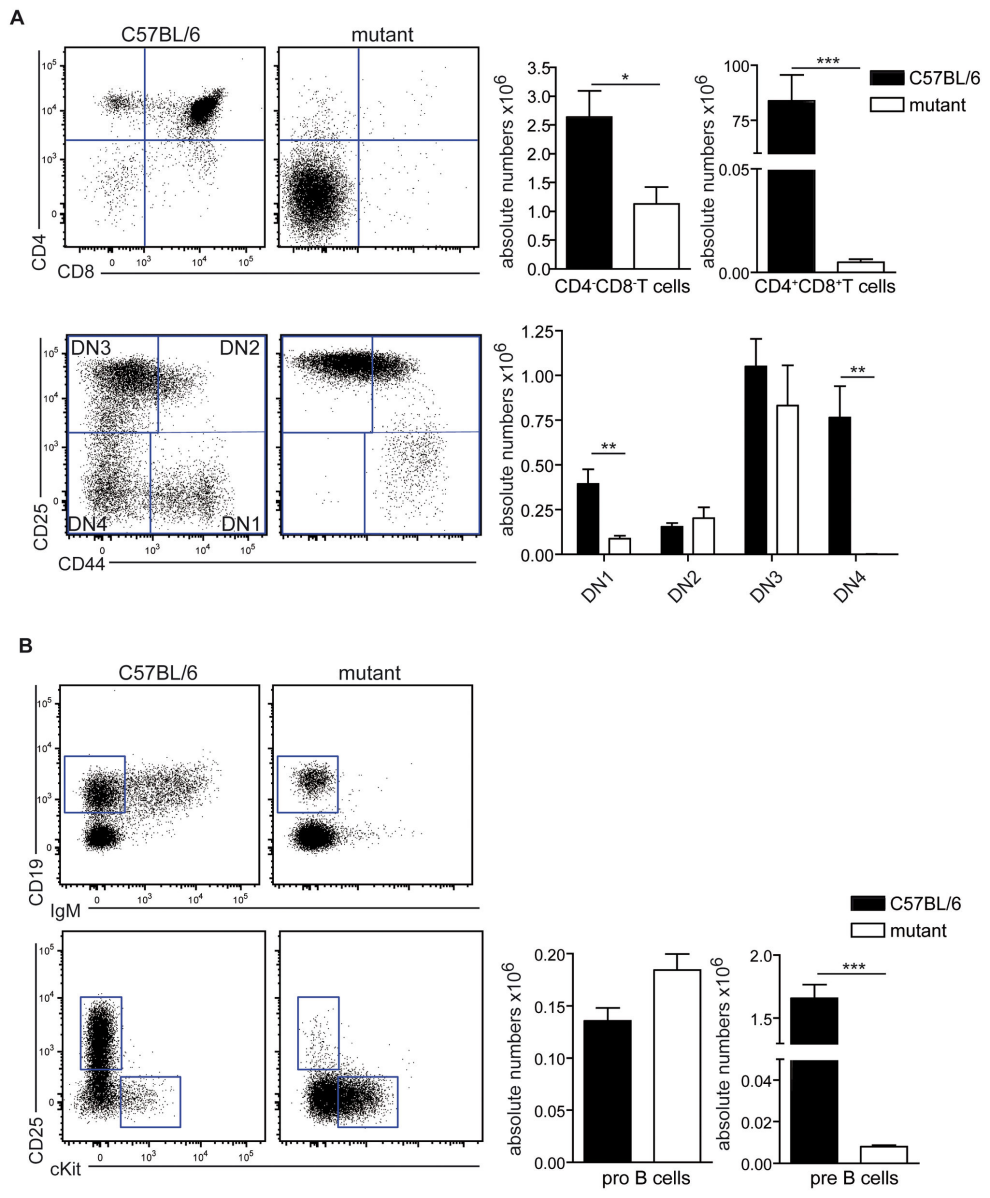
Characterization of T and B cell precursor development. (A) Thymocytes were stained with CD4 and CD8 to distinguish double negative (DN) and double positive (DP) thymocytes and with CD25 and CD44 to further divide the DN cell into DN1 (CD25^+^CD44^-^), DN2(CD25^+^CD44^+^), DN3(CD25^-^CD44^+^) and DN4(CD25^-^CD44^-^). Bar graphs represent mean number of live cells ± SD in the respective gate. Statistical significance was tested using Student’s t-test. (B) B cell precursors were analyzed in the bone marrow. CD19^+^IgM^-^ cells were further analyzed for the expression of cKit and CD25 to detect pro B cells (cKit ^+^ CD25^-^) and pre B cell (cKit^-^CD25^+^). FACS-plots and statistics are representative results of one of six experiments with 3 mice per group each. Bar graphs represent mean number of live cells ± SD in the respective gate. Statistical significance was tested using Student’s t-test. FACS-plots and statistics are representative results from one out of six experiments with similar results (n=3 mice per group).

To further characterize the influence of the novel mutation on T and B cell development, precursors were analyzed in thymus (Figure 4A) and bone marrow (Figure 4B). Thymocytes were almost exclusively double negative (DN) for CD4 and CD8 (Figure 4A) with hardly any cells reaching the double positive (DP) stage. These DN thymocytes were arrested in their development at the CD44^+^CD25^-^ ("DN3"), as DN4 cells were lacking completely. We also observed a decreased number of DN1 cells in Artemis-mutant mice (Figure 4A). The development of B cells was analyzed using CD25 and cKit to discriminate cKit^+^ pro-B cells and CD25^+^ pre-B cells (Figure 4B). Equal amounts of pro-B cells were found in wt and mutant mice, whereas pre-B cells were virtually absent in mutant mice, indicating that the *Dclre1c*-mutation blocked B cell development at the pro-B cells stage confirming the phenotype of previously published *Dclre1c*-knockouts [23,24,30]. 

## Discussion

We discovered a new T^-^B^-^NK^+^SCID mouse strain that lacked CD4^+^ and CD8^+^ T cells as well as B cells, but still generated normal numbers of NK cells as well as other cell types. Breeding the mutant mouse strain with healthy C57BL/6 or C3H animals showed that the phenotype was inherited in a recessive fashion, which is the way normal SCID mutations are inherited.

After a cross of the mutant mice with C3H and a subsequent intercross the phenotype was still unaltered in severity and occurred at the same frequency as on a pure C57BL/6 background (Figure 2A). This finding excluded the possibility of strain specific factors influencing the phenotype and argued for a monogenic trait. This breeding also allowed us to narrow down the deleted genomic region to a 42 Mbp in the p-region of chromosome 2 containing 649 genes. With *Dclre1c* the list of genes also included one gene known to cause SCID in knockout mice [23,31] as well as in humans [32]. By next-generation sequencing we could identify a deletion of exons 10 and 11 of the *Dclre1c* gene. Further sequence analyses identified a deletion between positions 18401 and 22436 of *Dclre1c* including exons 10 and 11 plus the additional loss of some of the introns surrounding these exons. A genomic deletion of comparable size has never before been described for *Dclre1c* in mice, while several such cases have been reported in humans [13,29,32-35]. The deletions in humans can be classified into two groups. In the first group Exons 1 to 3 or 4 were deleted by the recombination of these exons with a homologous sequence of a *Dclre1c* pseudo-gene 61.2 Kbp upstream of *Dclre1*c [32]. This mechanism cannot be responsible in mouse since a *Dclre1*c pseudo-gene does not exist in this species [32]. The second group contains deletions of exons downstream of exon 5, hence the region also affected in the novel mouse strain [29,32].

A possible mechanism for genomic deletions is the recombination of retrotransposable elements. This mechanism is responsible for most of the deletions in the 3´ part of the human *Dclre1c* gene [32,34]. For this to occur two homologous sequences have to be oriented identically on the same chromosome. A homologous recombination between the two sequences would then result in the deletion of the DNA in between. There are several retrotransposable elements in the *Dclre1c* gene, but not two identical regions flank the sequence deleted in this case. Hence, it is unclear whether retrotransposons played a role in the deletion of exon 10 and 11 and the mechanism responsible for the deletion of remains elusive.

The Artemis protein belongs to the large superfamily of metallo-β-lactamases [36]. The most N-terminal part is the metallo-β-lactamase domain containing most of the conserved Asp- and His-residues. The first domain is followed by the β-CASP domain containing the remaining two conserved Asp- and His-residues important for metal-ion coordination [36,37].

The C-terminal part of the protein does not share sequence homology with other proteins of the metallo-β-lactamase superfamily. The deletion of exons 10 and 11 results in an in frame sequence with amino acids 260 to 325 missing. The missing amino acids also contain His319, which plays a role in coordinating divalent metal ions essential for the biological functions of Artemis. The loss of this amino acid alone would be sufficient to abolish Artemis-activity in vivo [37,38]. On the other hand, it is not very likely that a protein would fold correctly with such a big amount of its amino acids missing.

The phenotype observed in the novel mouse mutant exactly phenocopies what is expected of a *Dclre1c* null-allele. The block in T and B cell development occurs at the transition from pro- to pre-B cells and from DN3 to DN4 T cells respectively, which is the time point at which the pre-TCR or pre-BCR would be expressed for the first time [39,40]. This signal is needed for the progenitors to progress in their development [41,42]. Considering the inability to successfully perform V(D) J rearrangements and the resulting lack of signaling through pre-antigenic receptor, B and T cell development is arrested at this stage. We also observed a reduced number of DN1 cells in the thymi of Artemis-mutant mice. This phenomenon had already been remarked previously in RAG-deficient mice [43], which also have a DN3 to DN4 transition blockade. There it was attributed to lack of development of thymic stromal [44,45] and endothelial cells [46], which would not allow normal numbers of DN1 cells to develop. As the size of thymi from Artemis-mutant mice also was considerable smaller as compared to wild type animals, the mechanism for reduction of DN1 cells might be the same.

A patient with a deletion of exons 10-12 showed a similar T^-^B^-^NK^+^ phenotype, which in this case could be rescued by bone marrow transplantation [29]. The same phenotype as in the novel mouse mutant could also be observed in mice with targeted deletions of *Dclre1c* [47], however, the complete block in B and T cell development was restricted only to mice of C57BL/6-, but not those of 129/SvJ-background [24]. It has been proposed that strain-specific factors can compensate in part for *Dclre1c*-deficiency. In our experiments no leakiness was observed on C57BL/6 or mixed background.

Our mouse model represents a spontaneously occurring mutation that faithfully resembles the human RS-SCID phenotype and may be a valuable tool to further investigate the mechanisms of V(D) J-rearrangement, SCID-disease as well as putative therapeutic treatment.
